# Bone Effect and Safety of One-Year Denosumab Therapy in a Cohort of Renal Transplanted Patients: An Observational Monocentric Study

**DOI:** 10.3390/jcm10091989

**Published:** 2021-05-06

**Authors:** Carlo Alfieri, Valentina Binda, Silvia Malvica, Donata Cresseri, Mariarosaria Campise, Maria Teresa Gandolfo, Anna Regalia, Deborah Mattinzoli, Silvia Armelloni, Evaldo Favi, Paolo Molinari, Piergiorgio Messa

**Affiliations:** 1 Unit of Nephrology, Dialysis and Renal Transplantation, Fondazione IRCCS Ca’ Granda Ospedale Maggiore Policlinico, 20122 Milan, Italy; valentina.binda@policlinico.mi.it (V.B.); silvia.malvica@unimi.it (S.M.); donata.cresseri@policlinico.mi.it (D.C.); maria.campise@policlinico.mi.it (M.C.); mariateresa.gandolfo@policlinico.mi.it (M.T.G.); anna.regalia@policlinico.mi.it (A.R.); deborah.mattinzoli@policlinico.mi.it (D.M.); silvia.armelloni@policlinico.mi.it (S.A.); paolo.molinari1@unimi.it (P.M.); piergiorgio.messa@unimi.it (P.M.); 2 Department of Clinical Sciences and Community Health, University of Milan, 20122 Milan, Italy; evaldo.favi@unimi.it; 3 Specialization School of Nephrology and Dialysis, University of Milan, 20122 Milan, Italy; 4 Renal Transplantation Unit, Fondazione IRCCS Ca’ Granda Ospedale Maggiore Policlinico, 20122 Milan, Italy

**Keywords:** denosumab, kidney transplantation, CKDMBD, vertebral fractures

## Abstract

In 32-kidney transplanted patients (KTxps), the safety and the effects on BMD and mineral metabolism (MM) of one-year treatment with denosumab (DB) were studied. Femoral and vertebral BMD and T-score, FRAX score and vertebral fractures (sVF) before (T0) and after 12 months (T12) of treatment were measured. MM, renal parameters, hypocalcemic episodes (HpCa), urinary tract infections (UTI), major graft and KTxps outcomes were monitored. The cohort was composed mainly of females, *n* = 21. We had 29 KTxps on steroid therapy and 22 KTxps on vitamin D supplementation. At T0, 25 and 7 KTxps had femoral osteoporosis (F-OPS) and osteopenia (F-OPS), respectively. Twenty-three and six KTxps had vertebral osteoporosis (V-OPS) and osteopenia (V-OPS), respectively. Seventeen KTxps had sVF. At T12, T-score increased at femoral and vertebral sites (*p* = 0.05, *p* = 0.008). The prevalence of F-OPS and V-OPS reduced from 78% to 69% and from 72% to 50%, respectively. Twenty-five KTxps ameliorated FRAX score and two KTxps had novel sVF. At T12, a slight reduction of Ca was present, without HpCa. Four KTxps had UTI. No graft rejections, loss of graft or deaths were reported. Our preliminary results show a good efficacy and safety of DB in KTxps. Longer and randomized studies involving more KTxps might elucidate the possible primary role of DB in the treatment of bone disorders in KTxps.

## 1. Introduction

Chronic kidney disease (CKD) is now a serious problem because of the progressive rise of its incidence and prevalence worldwide [1]. Among the several complications present in CKD patients, the alterations of bone and mineral metabolism (MM) have a strong impact. Several changes in bone structure and progressive disarrangement in MM homeostasis occur progressively in CKD patients and cause the insurgence of vascular calcifications, cardiovascular events and bone fractures [2].

Kidney transplantation (KTx) is considered the best option for patients affected by CKD. Compared to dialyzed patients, KTx patients (KTxps) have globally better life expectancy and better cardiovascular and global outcomes [3]. Nevertheless, KTx is not able to completely solve the metabolic disorders developed during CKD. In addition, some specificities of KTxps exert additional effects on MM and bone homeostasis [4,5]. KTxps have, for these reasons, a higher fracture risk than the general population [6]. Post KTx bone loss and fractures incidence have their highest degree during the 12 months after KTx, but a progressive bone loss is reported during the entire life of the graft [7]. The principal therapeutic options for the treatment of bone anomalies in KTxps involve vitamin D, calcium supplements and, when indicated, bisphosphonates [8]. The concerns about bisphosphonate prescription in KTxps, mostly related to their potential nephrotoxicity and the limited data on their efficacy in preventing novel fractures, have limited the use of these drugs, making the management of bone disorders still unsatisfactory in KTxps [9]. Denosumab (DB) is a fully human monoclonal antibody directed against RANKL that inhibits the osteoclast activity resulting in a progressive decrease of bone resorption [10]. In the general population, DB is now used as a valid alternative to bisphosphonate in preventing osteoporosis and bone fractures [11]. Unfortunately, data concerning its role in KTxps are still limited. The aim of our study was to evaluate, in a cohort of KTxps who underwent a one-year treatment with DB: (1) the evolution of femoral and vertebral bone mineral bone density (BMD); (2) the effect on FRAX score and on the development of novel spontaneous vertebral fractures (sVF); (3) the modifications of the renal function and MM parameters; and (4) the safety of DB therapy.

## 2. Materials and Methods

### 2.1. Study Design

In our study, we evaluated prospectively, during the first year of treatment with DB, 32 KTxps (*M* = 11; median age 62 (58;69) years) followed up in our department. All patients studied were considered eligible to receive DB therapy by the presence of at least one of the following conditions: (1) sVF documented by X-ray; (2) femoral neck and/or vertebral osteoporosis; and (3) intolerance, long time treatment or contraindications to bisphosphonate therapy. In addition, only patients with basal Ca > 9.2 mg/dL and iPTH > 35 ng/dL were recruited.

After the initiation of DB, administered at the dosage of 60 mg every six months, all KTxps were followed regularly at our out-patient clinic for the whole period of observation and were treated in accordance with their clinical needs.

### 2.2. Instrumental Evaluations

Bone mass density was estimated at the proximal femur and at the lumbar spine (L1–L4) at T0 and T12 by means of dual energy X-ray absorptiometry (DEXA) as areal bone mineral density (BMD, g/cm^2^). According to the World Health Organization (WHO) criteria, we defined BMD with a T-score above 1 SD as not pathologic. A T-score between −1.0 and −2.5 SD was classified as osteopenia, whereas a T-score below −2.5 SD was defined as osteoporosis [12].

Vertebral fractures were evaluated by means of complete lumbar X-ray at T0 and T12 by means of Genant classification [13].

### 2.3. FRAX Score Evaluation

The FRAX score (including age, sex, body weight, height, history of prior osteoporotic fracture, parental history of hip fragility fracture, current smoking, arthritis, alcohol consumption >3 units/day and T-score) was calculated at T0 and T12 using the tool for Italy provided on the FRAX website [14].

### 2.4. Biochemical Evaluations

Data about biochemical analyses were digitally recorded from the documents presented by each patient at the out-clinic visits.

Briefly, the following parameters were recorded at pre-determined timepoints:

Before DB initiation, not more than one month (T0): serum creatinine (sCr), urea, Calcium (Ca), Phosphorus (P), parathormone (PTH), native vitamin D (25OHD), active vitamin D (1-25OHD), alkaline phosphatase (ALP), Magnesium (Mg) and daily urinary protein excretion (Prot-U);

One month after the start of DB therapy (T1): sCr and Ca;

Three months after the start of DB therapy (T3): sCr, Ca, P and PTH;

Six months after the start of DB therapy (T6): sCr, urea, Ca, P, PTH, 25OHD, ALP and Prot-U;

Twelve months after the start of DB therapy (T12): serum creatinine (sCr), urea, Ca, P, PTH, 25OHD, 1-25OHD, ALP, Mg and Prot-U.

PTH was measured by DiaSorin LIAISON^®^ kit. 25OHD levels were determined by enzyme-immunoassay (Kit EIA AC-57FI, immunodiagnostic system Boldon, UK), using a highly specific sheep 25OHD antibody and enzyme (horseradish peroxidase) labeled avidin. All other biochemical parameters were evaluated according to routine methodology used at the central laboratory of our Institution. All biochemical results were digitally recorded.

### 2.5. Clinical Events

During the follow up time, we evaluated the insurgence of the following clinical events:

- Hypocalcemia (HpCa): defined as a total serum Ca concentration < 8.0 mg/dl in the presence of normal plasma protein concentrations
- Urinary tract infections (UTI): defined by the presence of urinary symptoms associated to significative white blood cells (WBC > 50 m.f. 400×) and by the presence of positive urine-culture

In addition, biopsy proven graft rejection, graft failure and KTxps deaths were recorded.

### 2.6. Statistical Analyses

In statistical analyses, continuous variables were expressed as median value and interquartile range (25%;75%) and were log transformed if they had a skewed distribution.

Differences among groups were determined by Paired sign and Mann–Whitney, where indicated. Differences among percentages were determined by X_2_ test.

Statistical analyses were performed using software SPSS version 20^®^ and significance was set for *p* values < 0.05.

## 3. Results

### 3.1. Cohort Characteristics

Our cohort was composed mainly of females, and the median age of the overall cohort was 62 (58;69) years. As reported in Table 1, 24 KTxps underwent hemodialysis before KTx, and the median dialysis vintage was 53 (26;136). Glomerulonephritis was the main reason for end stage renal disease. Half of the patients received steroid therapy before KTx. Ninety-six percent of KTxps were transplanted by a deceased donor. Considering the overall cohort, DB was started after a median time of KTx of 144 (59;232) months.

In Table 2, the principal characteristics of the immunosuppressive and MM related therapies are reported. At both T0 and T12, the immunosuppressive therapy was composed principally by calcineurin inhibitors, mycophenolate and steroids, with no differences between the two timepoints considered. Steroid therapy was prescribed in 90% of KTxps, at a dosage of 5 (2.5;5) mg/day.

In 54% of KTxps, previous therapy with bisphosphonate was reported (Bisp+). The suspension of the drug was mainly related to the long-time course and/or the development of significant contraindications.

More than half of KTxps were receiving at T0 25OHD supplementation. During the follow up time, in one patient, 25OHD supplementation was started, whereas, in another case, Calcitriol was added to 25OHD supplementation. The mean dosage/week of 25OHD supplementation was similar between T0 and T12.

### 3.2. DEXA and Lumbar X-ray Evaluations

In Table 3, the principal findings concerning femoral and vertebral DEXA and lumbar X-Ray examinations are summarized.

At T0, normal bone density was found only in three KTxps, at vertebral level. The most prevalent finding was femoral osteoporosis (F-OPS), present in 78% of KTxps. At femoral level, F-BMD was 0.53 (0.48;0.60) g/cm^2^ and median T-score was −3.0 (−3.5;−2.5). Vertebral osteoporosis (V-OPS) was found in 75% of KTxps, and V-BMD and T-score were, respectively, 0.72 (0.65;0.87) g/cm^2^ and −3.0 (−3.7;−1.9). To investigate the possible effect on BMD improvement of a previous therapy with bisphosphonates, a sub-analysis considering Bisp+ and patients with no history of bisphosphonate before the DB treatment (Bisp−) separately was performed. No significant impact of previous bisphosphonate was evidenced at femoral and vertebral levels both at baseline and during the time of follow up.

After 12 months of DM therapy, global modifications in DEXA were found. At both femoral and vertebral level, the T-score significantly improved: femoral T-score reached −2.8 (−3.5;−2.4) (*p* = 0.05 vs. T0), whereas vertebral T-score −2.6 (−3.0;−1.6) (*p* = 0.008 vs. T0).

**Figure 1:**
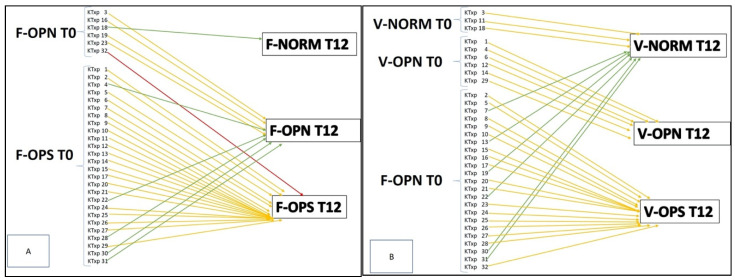
General distribution and DEXA class modifications at femoral (**A**) and vertebral (**B**) level during the time of observation. F-OPN, femoral osteopenia; F-OPS, femoral osteoporosis; F-norm, femoral normal DEXA; V-OPN, vertebral osteopenia; V-OPS, vertebral osteoporosis; V-norm, vertebral normal DEXA; red lines, worsening of DEXA category; yellow lines, stability of DEXA category; green lines, amelioration of DEXA category.

As reported in Figure 1A, the increase of T-scores also resulted in a re-distribution of the KTxps among the DEXA groups. At femoral level, a significant reduction of the prevalence of osteoporosis (*p* = 0.001) was found. Five patients moved from osteoporosis to osteopenia. One patient ameliorated his status from osteopenia to normal femoral T-score, whereas, in one patient, a worsening to osteoporosis was found.

At vertebral level, a significant reduction of the prevalence of osteoporosis (*p* < 0.0001) was also found. In this case, six KTxps moved from osteoporosis, five KTxps to osteopenia and one case in the normal T-score values group. In addition, the three KTxps who showed normal T score values at T0 confirmed their result at T12 (Figure 1B).

A sub analysis considering 20 KTxps not included in the study (10 KTxps for femoral BMD and 10 other KTxps for vertebral BMD) who were treated with neither DB nor bisphosphonates was also performed.

Those patients were matched to those considered in the study for age, time of transplant, gender distribution and obviously femoral and lumbar BMD.

In femoral DEXA, we found not significant modifications at T0 and after one year in BMD (0.55 (0.54;0.58) vs. 0.55 (0.52;0.60), *p* = 0.54) and femoral T score (−2.8 (−3.1;−2.7) vs. −2.7 (−3.0;−2.4), *p* = 0.13). The same result was found at lumbar level for BMD (0.70 (0.65;0.77) vs. 0.70 (0.69;0.71), *p* = 0.71) and T score (−3.25 (−3.6;−2.5) vs. −3.15 (−3.4;−3.0), *p* = 0.27). Unfortunately, no data about sVF were available for those patients.

The treatment with DB resulted in a global modification of FRAX score in the overall cohort. In particular, a reduction of FRAX score was found in 78% of KTxps, with mean values of FRAX score at T0 of 29 ± 15 % and at T12 of 26 ± 15 % (*p* = 0.18). Accordingly, at the second vertebral X-ray evaluation, novel sVF were found only in 6% of KTxps. They already had sVF at T0.

### 3.3. Biochemical Evaluations

As reported in Table 4, renal functional parameters were similar between T0 and T12. Among MM evaluations, we found significant modifications only in Ca (9.60 (9.37;10.21) vs. 9.40 (8.98;9.83), *p* = 0.01), PTH (63 (36;86) vs. 115 (44;161), *p* = 0.009) and ALP (68 (61–90) vs. 51 (45;68), *p* = 0.002) levels. No significant differences were found in P, Mg, 25OHD and 1-25OH levels.

### 3.4. Clinical Outcomes

During the year of follow up, no symptomatic or asymptomatic HpCa episodes were reported. Four KTxps had UTI (mean time from DM initiation: 114 days) and required specific antibiotic treatments. Of note, those four KTxps (two of them symptomatic) had positive pathologic anamnesis for UTI, so a direct relation to DB therapy is not possible to demonstrate. In two KTxps, a hospitalization for UTI complications (sepsis) was required. No biopsy proven graft rejections were observed during the time of treatment and no graft loss or KTxps death was reported.

## 4. Discussion

In this monocentric, observational, prospective study, 32 KTxps underwent, for clinical indications, DB therapy.

KTxps have a high risk of bone loss, initially related to pre-KTx clinical history. Steroid therapy before KTx, basal nephropathy and dialysis vintage are certainly impacting factors. In our cohort, similar to the data reported in the few investigations concerning DB therapy in KTxps, half of KTxps received steroids before KTx, and the most prevalent basal nephropathy was glomerulonephritis [15].

Even if most of the research present in the literature has focused their interests on the effect of DB in “early transplanted KTxps”, osteopenia and osteoporosis are complications present also in long-term KTx [16]. For this reason, we decided to evaluate KTxps independently of their time of KTx.

Most of the KTxps evaluated in our study at baseline were receiving some MM specific therapies. In more than half of cases, native vitamin D was prescribed at baseline, and the levels of 25OHD were almost sufficient. Fifty-four percent of KTxps previously received bisphosphonate. In the few patients in which bisphosphonates were prescribed at T0, the principal reasons for the shift to DB were: (1) long-term bisphosphonate therapy (>3 years); and (2) presence of bisphosphonate related adverse events (gastrointestinal discomforts, suspected nephrotoxicity).

The initial aims of our study were to test the evolution of femoral and vertebral BMD and the incidence of novel sVF. At baseline, more than half of KTxps had F-OPS and V-OPS. Compared to the papers published by Brunova et al. in 2018 and by Bonani et al. in 2016, T0 F-Ts and V-Ts of our cohort were significantly worse [14,17]. This might be related to the longer time of transplant of our cohort. In addition, sVF were present in more than half of our cohort at T0. This result confirms the high prevalence of sVF in KTxps. Some data indicate a strong fracture risk, especially during the first 5–6 years after KTx, related to a higher need of steroids and stronger immunosuppressive therapy. In his paper, published in 2014, Sukumaran Nair indicated that fracture risk event rate is higher in the first year after KTx, but still present in the following years of KTx [18]. Some years before, Nikkel reported fracture events in 22.5% of the cohort of KTxps studied within five years of KTx [19]. Currently, however, the increasing attention to the fracture risk and the consequent use of immunosuppressive and MM schemes of therapy directed to prevent bone loss might have modified the fracture risk in KTxps.

After one year of therapy with DB, a global amelioration of BMD was observed at both femoral and vertebral levels. As shown in the results, the increase of BMD compared to the baseline was significant, especially at vertebral level. This result, after only one year of treatment, confirms the good effect of DB on osteoclastic resorption of trabecular structures already evidenced in the general population [20]. The beneficial effect of DB on BMD was confirmed also by the relative low incidence of novel sVF during the year of treatment. Of note, a global amelioration of FRAX score was also observed [21]. Novel sVF were found in only two KTxps at T12. The role of DB in preventing VF was explored by Cummings in the general population by means of the evaluation of DB effect on 7868 women affected by osteoporosis. In this study, DB reduced the risk of new radiographic vertebral and non-vertebral fractures compared to placebo [11]. Now, to our knowledge, our study is the first that evaluated the follow up of vertebral fractures in KTxps treated with DB. However, the beneficial effect of this therapy in CKD patients might assume a similar effect also in the presence of KTx [22]. Certainly, this point should be analyzed in greater depth by means of specific prospective trials. In any case, our results even if partially limited by the limited time of follow up might be a good prospect. Another point that should be analyzed in the future is the impact on sVF of DB discontinuation, potentially a burden in the general population by a severe bone turnover rebound and a rapid loss of BMD, resulting in a strong increase of sVF risk [23]. Some possible solutions both in the general population and in KTxps might be the administration of bisphosphonate, as proposed by some authors [24], or the increase of time between each DB administration, resulting in a permanent turnover of the bone metabolism (personal opinion).

In our study, we also evaluated the trend of renal functional and MM parameters. During the year of observation, all renal functional parameters remained stable, and no differences between T0 and T12 were found. A good level of renal safety has already been reported in the general population and in solid organ transplantation [25].

The baseline values of Ca showed a significant decrease during the year of treatment. However, despite Ca levels of our cohort being similar to those reported in other studies, in our cohort neither symptomatic nor asymptomatic hypocalcemic events were reported. This might reflect the effect of the strict monitoring scheduled in our cohort that permitted a prompt correction of Ca levels, needed in a small part of the cohort studied. This observation is substantially in line with previous findings [26]. The reduction in Ca levels, mostly related to the reduced osteoclast activity, might in part also explain the increase of PTH levels. A slight increase of PTH levels during one year of therapy is in line with previous reports. An increase of PTH in those patients might have some beneficial effects on bone structure, especially on cortical porosity, as already reported in general population [27,28]. However, a deeper evaluation of bone effects of this increase of PTH in KTx population might be the topic of future studies by means also of bone biopsies [29].

Infections are an important cause of mortality and morbidity during KTx [30]. In our study, we also evaluated the insurgence of UTI in KTxps treated with DB. Some experimental evidence reports a possible influence of DB, by means of the inhibition of RANK/RANKL pathways, in favoring UTI insurgence [30]. The relationship between infection risk and DB therapy in de novo KTxps was explored by Bonani et al. in 2017. In their work, with the aim to evaluate the incidence of infections (especially UTI and viral), the authors randomized 90 de novo KTxps to receive or standard therapy without DB or DB therapy. The incidence of UTI was higher in the DB group. Of note, the prevalence of severe infections (pyelonephritis and/or urosepsis) was not different in the two study arms. [31] The UTI observed in our cohort were reported during the follow up period only in patients with a previous positive anamnesis for UTI. The single arm design, the different definition of UTI and the longer time of KTx of our cohort make the comparison of our results with those presented by Bonani difficult. However, future studies, possibly multicentric and with a uniform definition of UTI, might better explore this important topic.

The impact of DB therapy was considered also in the prevalence of graft rejection and graft and KTxps survival. No biopsy proven KTx rejections were found in our cohort during the follow up time. All KTxps who started the DB therapy ended safely the first year of therapy. The safety of Denosumab on those outcomes has been explored recently [14]. In accordance with the evidence found in our study, a directly related higher risk of worse graft and KTxps outcomes in patients treated with DB is not reported in the literature.

The present paper presents some limitations. Undoubtedly, the monocentric design reduced significantly the size of the cohort studied, but it allowed a uniformity in the cohort identification and its follow up. In addition, unfortunately, it was not possible to perform some more specific dosages concerning bone remodeling markers that might have clarified better the efficacy of DB in our cohort. The absence of a control group might be considered a limit of the study. In any case, our study was designed to evaluate the efficacy and the potential adverse events of the drug therapy and can be considered an important starting point for future randomized research.

In conclusion, the experience of our center demonstrated a good bone efficacy and general safety of one-year DB therapy in KTxps.

Certainly, future longer and randomized studies, involving more KTxps, might elucidate the possible primary role of DB in the treatment of bone disorders in these patients.

## Figures and Tables

**Table 1:** Cohort characteristics.

Parameter	
Number of patients, *n*	32
Gender (M/F), *n*	11/21
Previous type of dialysis (HD/PD), *n*	24/8
Dialysis vintage (months)	53 (26;136)
Basal Nephropathy *n* (%) - Gnf - ADPKD - Secondary nephropathies - Other	13 (40) 6 (18) 5 (15) 8 (27)
History of steroid therapy before KTx *n* (%)	16 (50)
Kind of transplant *n* (%) (deceased donor/living donor)	31/1 (96/4)
Age at Denosumab initiation (years)	62 (58;69)
Time of KTx at Denosumab initiation (months)	144 (59;232)

Footnotes: HD, hemodialysis; PD, peritoneal dialysis; Gnf, glomerulonephritis; ADPKD, autosomal dominant polycystic kidney disease; KTx, kidney transplantation.

**Table 2:** Immunosuppressive, mineral metabolism and anti-hypertensive therapies at T1 and T12.

Drug	T0	T12
CyA/Tac/MMF-MPA/AZA/mTor inhibitor *N* (%)	12/17/19/3/3 (37/53/58/9/9)	11/17/17/4/4 (34/53/50/12/12)
Steroid therapy *N* (%) Daily steroids (mg)	29 (90) 5 (2.5;5)	29 (90) 5 (2.5;5)
Vitamin D therapy *N* (%) - No - Native vitamin D - Native + active vitamin D	10 (31) 20 (63) 2 (6)	8 (25) 21 (66) 3 (9)
Previous therapy with bisphosphonate *n* (%)	17 (54)	NA
Dosage of native vitamin D (μg/week)	75 (0;100)	75 (0;100)
Cinacalcet therapy *N* (%)	6 (18)	4 (12)
Calcium supplements *N* (%)	3 (9)	4 (12)

Footnotes: Cya, cyclosporine; Tac, Tacrolimus; MMF-MPA, mycophenolate: azathioprine.

**Table 3:** Densitometry and X-ray evaluation at the two timepoints.

Parameters	T0	T12	*p*
F-BMD (g/cm^2^)	0.53 (0.48;0.60)	0.56 (0.49;0.66)	**0.02**
F-T-score	−3.0 (−3.5;−2.5)	−2.8 (−3.5;−2.4)	**0.05**
V-BMD (g/cm^2^)	0.72 (0.65;0.87)	0.79 (0.71;0.92)	**0.01**
V-T-score	−3.0 (−3.7;−1.9)	−2.6 (−3.0;−1.6)	**0.008**
Femoral bone density *N* (%) - Norman bone density - Osteopenia - Osteoporosis	0 (0) 7 (22) 25 (78)	1 (4) 9 (28) 22 (69)	**0.001**
Vertebral bone density *N* (%) - Normal bone density - Osteopenia - Osteoporosis	3 (10) 6 (18) 23 (72)	4 (13) 12 (37) 16 (50)	**<0.001**
FRAX score (%) FRAX score amelioration *n* (%)	29 ± 15 * NA	26 ± 15 * 25 (78)	0.18 NA
Patients with X-ray sVF *N* (%) Patients with novel X-ray sVF *N* (%)	17 (53) NA	17 (53) 2 (6)	NA

Footnotes: BMD, bone mineral density; F-BMD, femoral BMD; V-BMD, vertebral BMD; F-T-score, T score measured at femoral level; V-T-score, T score measured at vertebral level; sVF, spontaneous vertebral fracture; bold format indicates statistical significance (*p* < 0.05); NA, not applicable; * mean ± standard deviation; X_2_ square tests, paired sign tests were used.

**Table 4:** Biochemical characteristics of the overall cohort at T1 and ad T12.

Parameters	T0	T1	T3	T6	T12	p *
s-Creatinine (mg/dL)	1.32 (0.96;1.78)	1.25 (1.0;1.80)	1.41 (1.13;1.80)	1.24 (0.92;1.60)	1.33 (0.97;1.72)	0.35
s-Urea (mg/dL)	62 (42;82)	NA	NA	57 (47;81)	61 (47;91)	0.15
Prot-U (g/24 h)	0.157 (0.12;0.31)	NA	NA	0.21 (0.13;0.35)	0.17 (0.11;0.34)	0.86
Ca (mg/dl)	9.60 (9.37;10.21)	9.60 (9.0;9.86)	9.79 (9.40;9.96)	9.53 (9.10;10.0)	9.40 (8.98;9.83)	**0.01**
P (mg/dL)	3.05 (2.60;3.40)	NA	2.70 (2.40;3.0)	2.90 (2.30;3.40)	2.85 (2.33;3.30)	0.06
PTH (ng/mL)	63 (36;86)	NA	119 (63;177)	101 (51;136)	115 (44;161)	**0.009**
ALP (U/dL)	68 (61;90)	NA	NA	53 (44;69)	51 (45;68)	**0.002**
25OHD (μg/dL)	26.7 (16.0;42.8)	NA	NA	31.5 (21.4;39.4)	32 (16;39.9)	0.35
1-25OHD (ng/L)	47.8 (36.1;61.1)	NA	NA	NA	37.9 (28.7;52.5)	0.69
Mg (mg/dL)	1.86 (1.70;2.09)	NA	NA	1.92 (1.75;2.08)	1.89 (1.68;2.07)	0.79

Footnotes: PTH, parathormone; Ca, calcium; P, phosphorus; ALP, alkaline phosphatase; Prot-U, daily urinary protein excretion; bold format indicates statistical significance (*p* < 0.05), where t-test, Mann–Whitney and Kruskal–Wallis test were used.; NA, not applicable; * T0 vs. T12.

## Data Availability

If needed, data are available in anonymous form.
